# Analysis of by high-throughput sequencing: *Helicobacter pylori* infection and salivary microbiome

**DOI:** 10.1186/s12903-020-01070-1

**Published:** 2020-03-20

**Authors:** Yingjie Ji, Xiao Liang, Hong Lu

**Affiliations:** Division of Gastroenterology and Hepatology, Key Laboratory of Gastroenterology and Hepatology, Ministry of Health, Renji Hospital, School of Medicine, Shanghai Jiao Tong University, Shanghai, China

**Keywords:** *Helicobacter pylori*, Salivary microbiota, High-throughput sequencing, 16S rDNA

## Abstract

**Background:**

There have been reports of *Helicobacter pylori* (*H. pylori*) in the oral cavity and it has been suggested that the oral cavity may be a reservoir for *H. pylori* reflux from the stomach. High-throughput sequencing was used to assess the structure and composition of oral microbiota communities in individuals with or without confirmed *H. pylori* infection.

**Methods:**

Saliva samples were obtained from 34 *H. pylori* infected and 24 *H. pylori* uninfected subjects. Bacterial genomic DNA was extracted and examined by sequencing by amplification of the 16S rDNA V3-V4 hypervariable regions followed by bioinformatics analysis. Saliva sampling was repeated from 22 of the 34 *H. pylori* infected subjects 2 months after *H. pylori* eradication.

**Results:**

High-quality sequences (2,812,659) clustered into 95,812 operational taxonomic units (OTUs; 97% identity). *H. pylori* was detected in the oral cavity in infected (12/34), uninfected (11/24) and eradicated (15/22) subjects by technique of high-throughput sequencing, occupying 0.0139% of the total sequences. Alpha diversity of *H. pylori* infected subjects was similar to that of uninfected subjects (Shannon: 1417.58 vs. 1393.60, *p* > 0.05, ACE: 1491.22 vs. 1465.97, *p* > 0.05, Chao 1: 1417.58 vs. 1393.60, *p* > 0.05, *t*-test). Eradication treatment decreased salivary bacterial diversity (Shannon, *p* = 0.015, ACE, *p* = 0.003, Chao 1, *p* = 0.002, *t*-test). Beta diversity analysis based on unweighted UniFrac distances showed that the salivary microbial community structure differed between *H. pylori* infected and uninfected subjects (PERMANOVAR, pseudo-F: 1.49, *p* = 0.033), as well as before and after *H. pylori* eradication (PERMANOVAR, pseudo-F: 3.34, *p* = 0.001). Using LEfSe analysis, 16 differentially abundant genera were defined between infected and uninfected subjects, 12 of which had a further alteration after successful eradication.

**Conclusions:**

Our study using high-throughput sequencing showed that *H. pylori* was present commonly in the oral cavity with no clear relation to *H. pylori* infection of the stomach. Both *H. pylori* infection and eradication therapy caused alterations in community and structure of the oral microbiota.

**Trial registration:**

clinicaltrials.gov, NCT03730766. Registered 2 Nov 2018 - Retrospectively registered, https://clinicaltrials.gov/ct2/show/ NCT03730766.

## Background

*H. pylori* is a Gram-negative bacterium that colonizes the human gastric epithelium. *H. pylori* infection is characterized by mucosal inflammation (gastritis) and may result in peptic ulcer disease or gastric adenocarcinoma [1]. *H. pylori* is transmitted between humans by a variety of routes including gastro-oral and fecal-oral mechanisms that include contaminated water and food. It has also been postulated that oral cavity may play a role in *H. pylori* transmission and possibly act as a reservoir [2]. For example, *H. pylori* has been detected in the oral cavity using the polymerase chain reaction (PCR). *H. pylori* has also been successfully cultured from saliva from individuals with positive results of both saliva *H. pylori* antigen test and *H. pylori* flagella test [3].

The oral cavity is one of the most complex and largest microbial habitats that harbors hundreds of different bacteria which play important roles in maintaining oral homeostasis and influencing the development of both oral and systematic diseases [4]. Many factors in the oral environment including intraoral pH and salivary iron concentration as well as expression of serum malondialdehyde and lipid profile have been reported to have significant relationships with oral microbial communities [5, 6]. Several recent studies detected the efficacy of antibiotics or drug composed of herbal on oral microbiol communities [7, 8]. However, there was few reports about *H. pylori* and its relationship with the microbial community structure in human saliva.

Currently, most oral bacteria species cannot be easily cultivated in vitro using traditional cultivation methods requiring the use of molecular biological techniques, such as checkerboard hybridization, microarray chips, and the quantitative real-time PCR to identify and classify the currently uncultivable bacteria [9]. However, many low-abundance bacteria species still cannot be detected using these approaches which impede the comprehensive and in-depth understanding of oral bacteria diversity. In this study, we used amplicon sequencing of 16S rDNA V3-V4 hypervariable regions to define the bacterial composition, abundance, and structure of salivary microbiome in people with and without active *H. pylori* infections. In addition, we also characterized the salivary biodiversity of a subgroup of subjects before and after the *H. pylori* eradication. As *H. pylori* passes through saliva, it is important to detect its expression in oral cavity as well as the efficacy of its infection and eradication on the oral microbiol communities.

## Methods

### Subjects and sample collections

The samples were collected in Renji Hospital of Shanghai Jiao Tong University, China, from August to November in 2018. A total of 58 subjects were recruited, including 34 subjects with *H. pylori* infection and 24 uninfected subjects. We first conducted a cross-sectional study of the salivary microbiota of 34 *H. pylori* infected and 24 uninfected subjects. A prospective study was then performed in a subgroup of 22 subjects with *H. pylori* infection who underwent salivary analysis both before and after successful *H. pylori* eradication.

All subjects received both endoscopy and ^13^C urea breath test (^13^C-UBT) before enrollment. The *H. pylori* infection status was confirmed by positive rapid urease test (RUT), histology and ^13^C-UBT. Absence of infection was defined as negative results for all tests (i.e., RUT, histology and ^13^C-UBT). *H. pylori* infected subjects received eradiation therapy consisting of esomeprazole 20 mg b.i.d., bismuth potassium citrate 600 mg b.i.d, amoxicillin 1000 mg t.i.d., and metronidazole 400 mg t.i.d. for 14 days. *H. pylori* eradication was confirmed by ^13^C-UBT at least 6 weeks after the end of treatment. Saliva samples were collected from 22 subjects both before and 2 months following successful *H. pylori* eradication. Subjects were characterized into four groups. *H. pylori* uninfected group (uninfected) (*n* = 24), *H. pylori* infected group (infected) (*n* = 34), and Pre-eradicated *H. pylori* infected group (pre-eradicated) (*n* = 22) and a successful eradicated group (eradicated) (*n* = 22). Inclusion criteria of subjects were: age of 20–65 years old male or female, with good oral hygiene (including brushing teeth twice a day for three minutes each) and with no bad eating habits [10]. Exclusion criteria included: 1) the presence of dental carious or any untreated cavitated carious lesions and oral abscesses, 2) periodontal disease or periodontal pockets ≥4 mm, 3) the use of antibiotics or proton pump inhibitors (PPIs) within 2 months before the study, 4) previous diagnosis of a serious systemic diseases (such as diabetes, hypertension or cardiopathy) or any diseases affecting oral health (such as Sjogren’s syndrome or any disease characterized by xerostomia), 5) pregnancy of breastfeeding, 6) specific eating habits or habits of eating specific food such as garlic or pepper and 7) smoking or alcohol drinking. The periodontal examinations were conducted by the same professional dentist in Renji Hospital of Shanghai Jiao Tong University, China, using the same diagnostic criteria. The detailed clinical parameters of the 58 subjects are shown in Additional file 1:Table S1.

### Salivary sampling

Sampling was performed in the morning 2 h after eating. Saliva samples were collected from each subject according to the Manual of Procedures for Human Microbiome Project (http://hmpdacc.org/resources/tools_protocols.php), with minor modifications. Approximately 3–4 ml of non-stimulated saliva was collected in two sterile, labeled 2 mL Eppendorf tubes, which were immediately placed on ice. Within 3 h of collection, samples were transported on ice and stored at − 80 °C until use.

### DNA extraction and sequencing

DNA was extracted from the saliva samples using the E.Z.N.A.® Soil DNA Kit (OMEGA, USA), following the manufacturer’s instructions, and stored at − 20 °C prior to further analysis. PCR amplification of the bacterial 16S rDNA hypervariable V3-V4 region was performed using the forward primer 338F (5′-ACTCCTACGGGAGGCAGCA-3′) and the reverse primer 806R (5′-GGACTACHVGGGTWTCTAAT-3′). Sample-specific 7-bp barcodes were incorporated into the primers for multiplex sequencing. Details of the barcodes are shown in Additional file 2: Table S2. PCR amplification were performed on an ABI 2720 instrument (ABI, USA) with an initial denaturation at 98 °C for 2 min, followed by 25 cycles of denaturation (15 s at 98 °C), annealing (30s at 55 °C), extension (30s at 72 °C), and ended with a final extension (5 min at 72 °C). A negative control was set during the process of PCR amplification to eliminate reagent contamination. PCR amplicons were purified with Agencourt AMPure Beads (Beckman Coulter, Indianapolis, IN) and quantified using the Quant-iT PicoGreen dsDNA Assay Kit (Invitrogen, Carlsbad, CA, USA). Equimolar concentrations of purified amplicons were pooled in equal amounts. Subsequently, the paired-end 2 × 300 bp sequencing plus 20% PhiX control DNA was performed on the Illumina MiSeq platform with MiSeq Reagent Kit v3 (Illumina, USA), following the vendor’s standard protocols.

### Sequence analysis

The Quantitative Insights Into Microbial Ecology (QIIME) pipeline was employed to process the sequencing data. Raw sequences were filtered to obtain high-quality sequences according to QIIME [11]. The low-quality sequences were filtered through following criteria: sequences that had a length of < 200 bp, sequences that had average Phred scores of < 25, sequences that contained ambiguous bases or homopolymeric stretches, and sequences that contained mononucleotide repeats of > 8 bp. The high-quality sequences were clustered into operational taxonomic units (OTUs) at 97% sequence identity by UCLUST [12]. The representative sequences selected from each OTU were classified taxonomically by BLAST searching against the Human Oral Microbiome Database (HOMD). OTU taxonomic classification was conducted by BLAST searching the representative sequences set against the HOMD using the best hit, with a BLAST e-value <1e-5. HOMD provides a detailed record of the type, metabolism, and pathogenicity of oral bacteria [13]. Then, an OTU table was further generated to record the OTU abundance of each sample and the taxonomic classification of these OTUs. Finally, to minimize the difference of sequencing depth across samples, the OTU table was modified by removing OTUs containing less than 0.001% of total sequences across all samples for further analysis [14].

### Bioinformatics and statistical analysis

Sequence data analyses were mainly performed using QIIME (version 1.8.0) and R packages (version 3.2.4). The alpha diversity analysis including Chao 1 richness estimator, Abundance-based Coverage Estimator (ACE) metric, Shannon diversity index, and Simpson index, were calculated at 97% identity using the QIIME [15]. Ranked abundance curves were generated to compare both the richness and evenness of OTUs among samples. The beta diversity analysis including Nonmetric Multidimensional Scaling (NMDS), and unweighted UniFrac distances based principal coordinate analysis (PCoA), were performed using the R package to evaluate the similarity among various bacterial communities [16]. The significance of differentiation of microbiota structure among groups was assessed by Adonis test [17]. The taxonomy compositions and abundances were visualized by MEGAN (version 6.6.7) software [18]. Linear discriminant analysis effect size (LEfSe) was used to compare the bacterial community structures between the samples from the patients with and without *H. pylori* infection, as well as before and after the eradication regimen, using the online Galaxy workflow framework (http://huttenhower.sph.harvard.edu/galaxy/) [19].

## Results

### Global sequencing data

The saliva samples were obtained from 34 *H. pylori* infected, 24 uninfected and 22 in 34 subjects 2 months after successful eradication therapy. A total of 2,812,659 high-quality sequences (representing 79% of the total sequences) were acquired from the 80 saliva samples, with an average of 35,158 sequences per sample (ranging from 19,210 to 44,310; Additional file 3: Table S3). The average sequence length was 445 bp, with the maximum length being 548 bp and the shortest length being 136 bp (Additional file 4: Figure S1). Clustering of all high-quality sequences at 97% identity by UCLUST resulted in 70,489 OTUs with an average of 4222 OTUs (ranging from 2170 to 5669), which were BLAST-searched against the HOMD for taxonomic assignments. After removing the low-credibility OTUs (together contributing only 6.7% of all sequences) and subsampling each sample to an equal sequencing depth of 19,210 reads per sample, a modified OTU table was obtained consisting of 95,812 OTUs with an average of 1198 OTUs per sample (ranging from 697 to 1584; Additional file 5: Table S4).

### Bacterial abundance and distribution

**Fig. 1:**
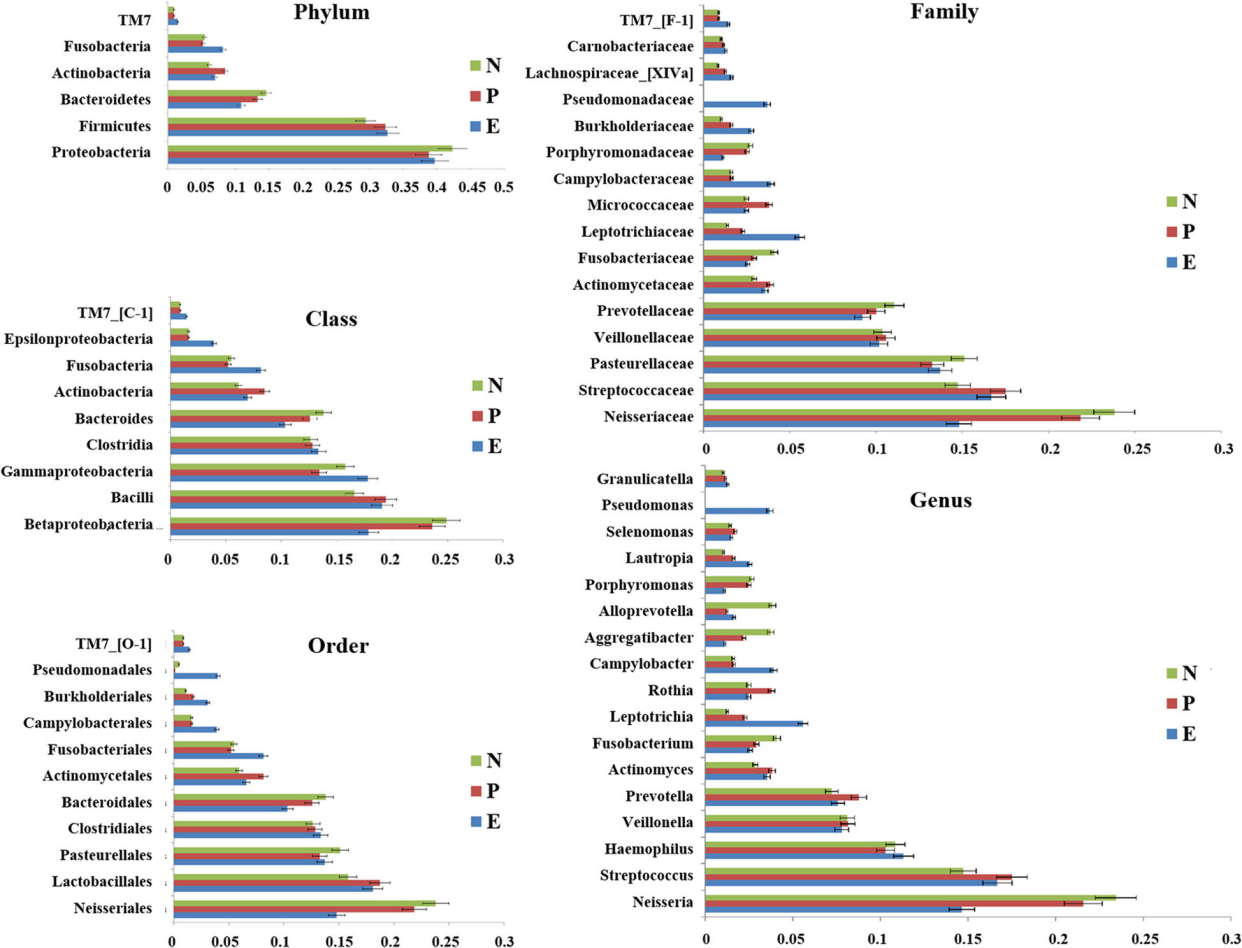
Distribution of the predominant bacteria at different taxonomic levels (phylum, class, order, family, and genus). The predominant taxa (> 1% relative abundance) in each level are shown. Red represent *P* group, green represent N group, and blue represent E group. N = uninfected group, P = infected group, E = eradicated group

The bacterial distribution was characterized in terms of the relative taxonomic abundances. A total of 11 phyla, 21 classes, 36 orders, 68 families, 138 genera and 440 species were detected in the saliva samples. The taxonomic distributions of the predominant bacteria (relative abundance > 1% of the total sequences) in subjects with and without *H. pylori* at different levels were shown in Fig. 1. The 6 most abundant phyla were *Proteobacteria* (40.1% of the total sequences), *Firmicutes* (31.6%), *Bacteroidetes* (13.0%), *Actinobacteria* (7.4%), *Fusobacteria* (6.1%), and *TM7* (1.0%), together accounting for 99.2% of the total sequences. At genus level, saliva microbiota was dominated by *Neisseria*, *Streptococcus*, *Haemophilus*, *Veillonella*, and *Prevotella*, with average relative abundances of 20.2, 16.5, 10.5, 8.0, and 8.0%, respectively. *Fusobacteria* was observed higher abundance in eradicated group on phyla level. *Leptotrichia, Campylobacter* and *Pseucomonas* expressed higher in eradicated group on genus level, while *Alloprevotella* and *Aggregatibacter* were observed higher abundance in uninfected group. The compositions in taxa of the microbial communities according to the tested sample groupings are provided in Additional file 6: Figure S2.

### Bacterial diversity analysis

The salivary microbiota richness, measured by numbers of observed OTUs, was similar in uninfected subjects and infected subjects (Additional file 7: Figure S3A). The alpha diversity indices of Chao1, ACE, Shannon, Inverse Simpson, Good’s coverage, and Simpson’s evenness are shown in Table 1. The Shannon diversity index was higher in uninfected subjects than in infected subjects, but there was no significant difference between groups by *t*-test (1417.58 vs. 1393.60, *p* > 0.05). Besides, the ACE richness index (1491.22 vs. 1465.97, *p* > 0.05), Chao 1 richness estimator (1417.58 vs. 1393.60, *p* > 0.05), and the Inverse Simpson diversity index (1.02 vs. 1.02, *p* > 0.05) was also higher in uninfected subjects, with no significant difference, indicating the similar bacterial diversity of *H. pylori* uninfected saliva compared to the infected subjects. Good’s coverage estimator for each group was over 98%, indicating that the current sequencing depth was sufficient to saturate the bacterial diversity of saliva. In addition, Simpson’s evenness index indicated that the bacterial-community distribution in two groups was uneven, which was also observed in the rank-abundance curve (Additional file 8: Figure S4).

**Table 1:** Alpha diversity indices for saliva bacteria in each group at 97% identity

Group	Chao1		ACE		Shannon		Inverse Simpson		Coverage		Simpsoneven	
	Mean	SD	Mean	SD	Mean	SD	Mean	SD	Mean	SD	Mean	SD
**N**	1417.58	195.47	1491.22	210.41	7.94	0.36	1.02	0.01	0.98	0.01	0.05	0.02
**P**	1393.60	182.57	1465.97	196.06	7.89	0.42	1.02	0.01	0.98	0.00	0.05	0.01

*SD* Standard Deviation. *N* uninfected group, *P* infected group

No statistically significant difference was observed in all index among N group and P group (*p* > 0.05, Student’s t-test)

### Bacterial community structures

**Fig. 2:**
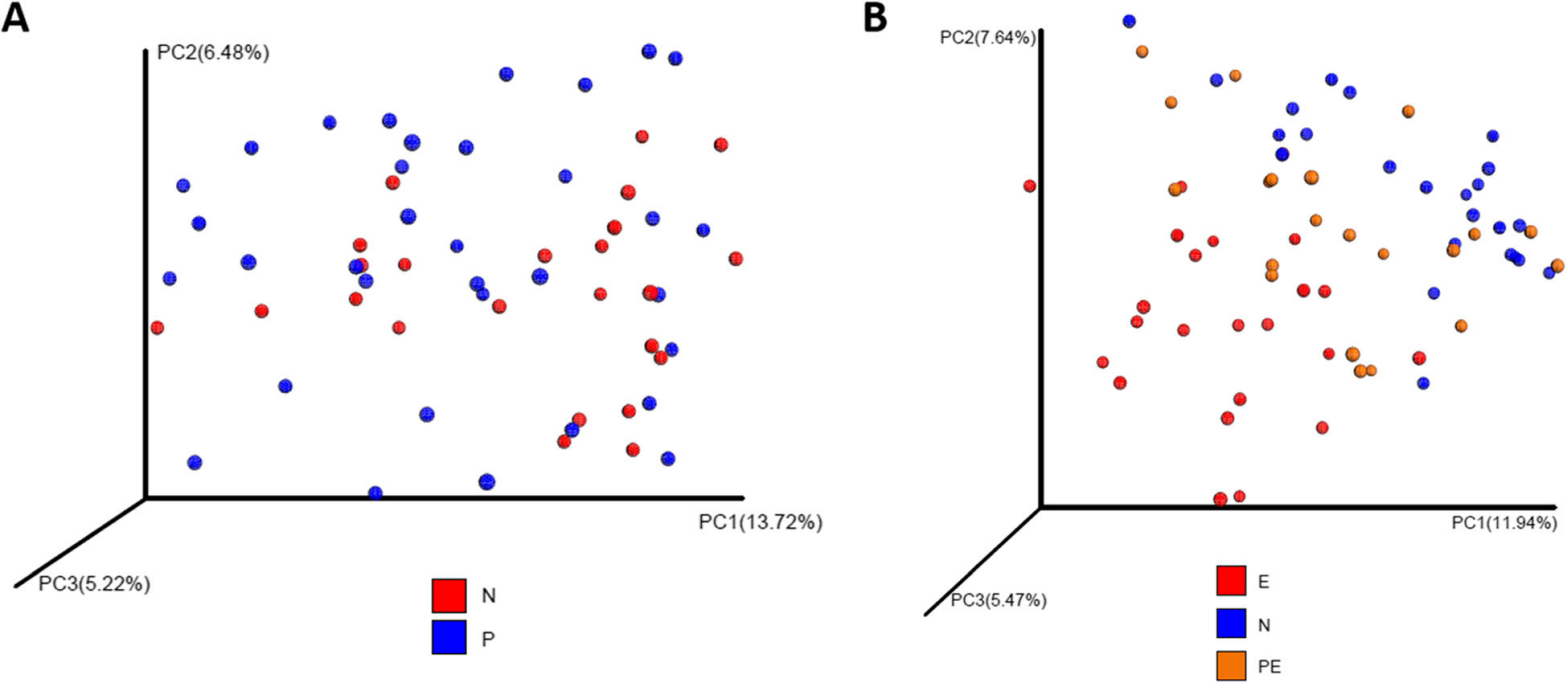
Principal coordinate analysis (PCoA) of unweighted UniFrac analysis. **a.** PCoA analysis demonstrated that subjects of *P* group were significantly different from N group (PERMANOVAR, pseudo-F: 1.49, *p* = 0.033). N = uninfected group, P = infected group. **b.** PCoA analysis showed that the overall microbial composition showed significant difference between PE and E group (PERMANOVAR, pseudo-F: 3.34, *p* = 0.001). E = eradicated group, N = uninfected group, PE = pre-eradicated group

To gain insights into the similarities in the bacterial community structures among uninfected and infected subjects, PCoA of beta diversity analysis was performed based on the unweighted UniFrac distances, which demonstrated different community structures among two groups (PERMANOVAR, pseudo-F: 1.49, *p* = 0.033). As shown in Fig. 2a, the overall microbial composition of infected subjects deviated from that of uninfected subjects. Furthermore, the results of NMDS based on the genus level classification exhibited clear segregations in community structures among groups (Additional file 9: Figure S5A).

### Differential microbiota compositions

**Fig. 3:**
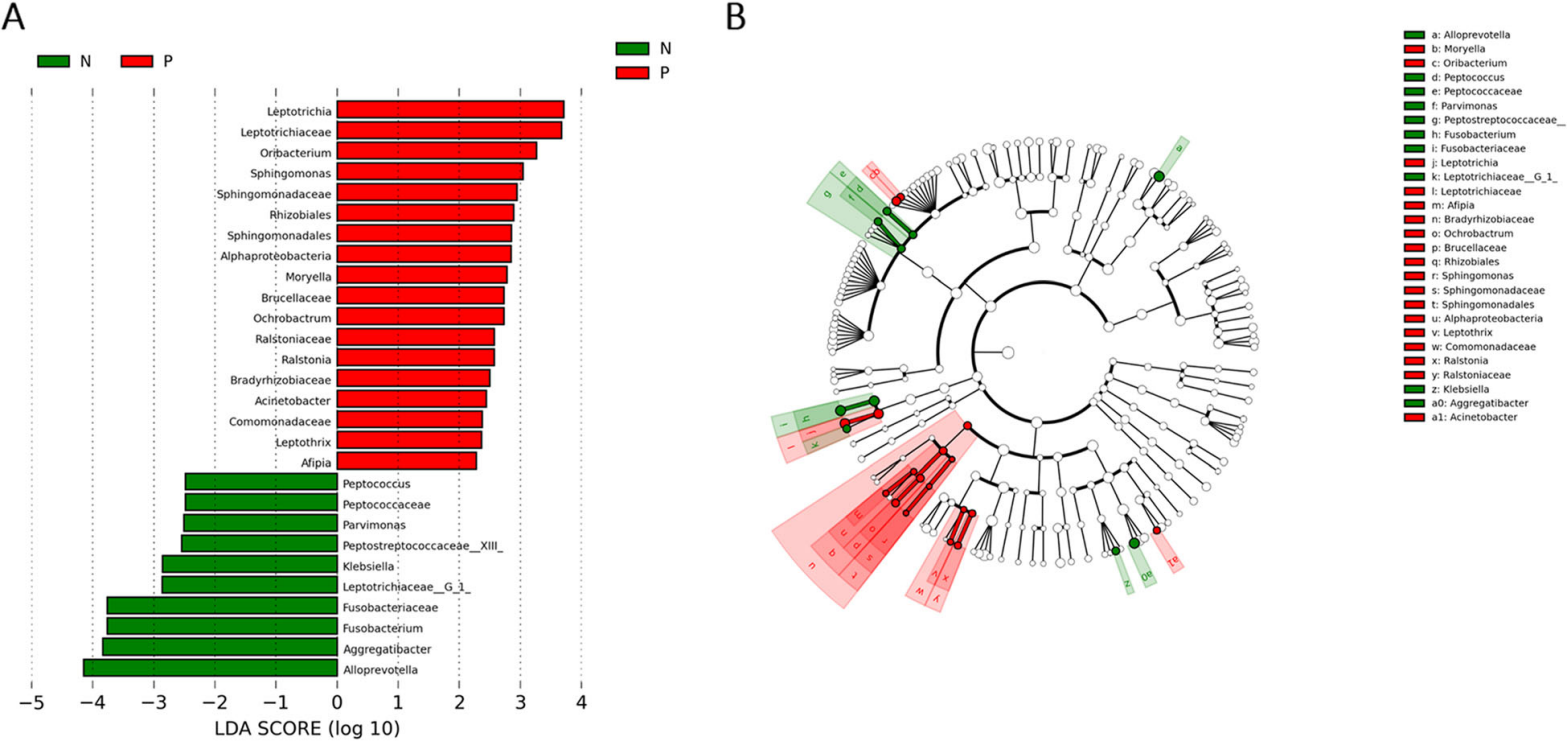
Comparison of microbial variations at the genus level, using the LEfSe online tool**. a.** Histogram of the LDA scores for differentially abundant features among groups. The threshold on the logarithmic LDA score for discriminative features was set to 2.0. N = uninfected group, P = infected group. **b.** Cladogram for taxonomic representation of significantly differences among groups. Differences are represented in the color of the most abundant taxa (red indicating *P* group, green indicating N group, and white indicating non-significant). N = uninfected group, P = infected group

There were significant differences in the community compositions among two groups. As shown in Fig. 3, a cladogram representation of significantly different taxa among groups was performed by LEfSe. The microbial composition was significantly different at the genus level, with 16 significantly different genera among the two groups. *Acinetobacter*, *Ralstonia*, *Leptothrix*, *Sphingomonas*, *Ochrobactrum*, *Afipia*, *Leptotrichia*, *Oribacterium*, and *Moryella* exhibited a relatively higher abundance in infected subjects, and can be considered *H. pylori*-enriched genera. *Alloprevotella*, *Aggregatibacter*, *Klebsiella*, *Leptotrichlaceae__G_1_*, *Fusobacterium*, *Parvimonas*, and *Peptococcus* were relatively more abundant in uninfected subjects, which could be considered to be decreased in the infected group. These higher or lower expressed genera in infected subjects can be considered as *H. pylori*-associated genera (LAD > 2, *p* < 0.05).

### Eradication therapy for *H. pylori* partially changed salivary microbiota

To determine the effects of *H. pylori* eradication therapy on salivary microbial composition, saliva samples from a subgroup of *H. pylori* infected subjects (*n* = 22), were collected before (pre-eradicated group) and 2 months after treatment; saliva collected after successful eradication were classified into the eradicated group. The within-individual diversity in the samples from eradicated group was lower than pre-eradicated group (Additional file 7: Figure S3B). The Shannon diversity index, ACE richness index, and Chao 1 richness estimator were higher in pre-eradicated subjects than in eradicated subjects, with a significant difference between groups by *t*-test (Shannon *p* = 0.015, ACE *p* = 0.003, Chao 1 *p* = 0.002), indicating significant alteration in the within-individual diversity in samples from eradicated subjects compared to their baseline samples (Table 2).

**Table 2:** Alpha diversity indices for saliva bacteria in each group at 97% identity

Group	Chao1		ACE		Shannon		Inverse Simpson		Coverage		Simpsoneven	
	Mean	SD	Mean	SD	Mean	SD	Mean	SD	Mean	SD	Mean	SD
**PE**	1350.56^*^	200.94	1421.26^*^	215.43	7.83^*^	0.49	1.02	0.01	0.98	0.00	0.05	0.02
**E**	1143.95^*^	215.08	1206.85^*^	234.63	7.44^*^	0.53	1.03	0.02	0.98	0.00	0.05	0.02

*SD* Standard Deviation. Asterisk indicates significant difference (*p* < 0.05, Student’s t-test). *PE* pre-eradicated group, *E* eradicated group

^*^Chao 1, ^*^ACE, and ^*^Shannon index between PE and E was statistically significant different (*p* < 0.05)

The beta diversity using unweighted UniFrac showed significant differences in the overall microbial composition between pre-eradicated and eradicated groups (PERMANOVAR, pseudo-F: 3.34, *p* = 0.001) (Fig. 2b). The NMDS also exhibited clear segregations in community structures among groups (Additional file 9:Figure S5B).

The relative difference of *H. pylori*-associated taxa was compared before and after eradication by LEFse analysis (Additional file 10:Figure S6). Among the *H. pylori*-enriched genera, *Ralstonia*, *Leptotrichia*, *Sphingomonas*, *Leptothrix*, *Oribacterium*, and *Acinetobacter* increased after the eradication, while *Ochrobactrum* decreased after the successful eradication (*p* < 0.05, paired Wilcoxon rank-sum test). Of the genera that decreased in infected subjects, *Alloprevotella*, *Aggregatibacter*, *Leptotrichlaceae__G_1_*, *Parvimonas*, and *Fusobacterium* decreased after the eradication (*p* < 0.05, paired Wilcoxon rank-sum test). Besides, we found that at phyla level, *Fusobacteria* increased after *H. pylori* eradication.

### *H. pylori* in the oral cavity

**Fig. 4:**
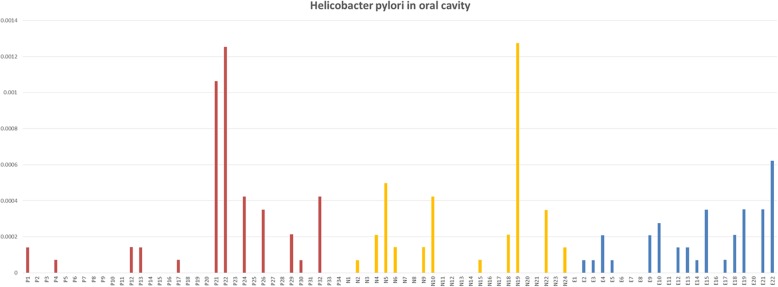
*H. pylori* in oral cavity of three groups. Red represent *P* group, yellow represent N group, and blue represent E group. N = uninfected group, P = infected group, E = eradicated group

*H. pylori* was detected in 38 out of 80 saliva samples, regardless of the *H. pylori* status in stomach, occupying 0.0139% of all the total sequences. (Fig. 4). 12 of the 34 subjects in infected subjects (35.3%), 11 of 24 subjects in the uninfected group (45.8%), and 15 of 22 subjects in the eradicated group (68.2%) were found to possess *H. pylori* in the oral cavity, respectively, (*p* = 0.054). The *H. pylori* signature was present in the saliva of subjects with negative ^13^C-UBT, RUT, and in *H. pylori* infected individuals after successful *H. pylori* eradication. We’ve also compared the prevalence of *H. pylori* both before and after *H. pylori* eradication. Pre-eradiation 7 subjects has positive saliva and 15 were negative. After successful eradication, *H. pylori* was still detected from saliva in 6 of the 7 positive subjects. Interestingly, 9 in 15 subjects who were *H. pylori* negative before eradication had it detected post *H. pylori* eradication whereas 6 remained free of *H. pylori* after eradication.

## Discussion

In this study, using the technology of high-throughput sequencing, we found *H. pylori* exit in the oral cavity of infected (12/34), uninfected (11/24) and successful eradicated (15/22) subjects, composing 0.0139% of the total sequences.

A few studies have shown that *H. pylori* can be spread by oral-oral (or fecal-oral) way [2]. Feeding, kissing, and tableware shared play the important role in the transmission. Previous studies have detected the presence of *H. pylori* in saliva [3]. Bacteria in plaque adhere to the gums, which are relatively fixed. It is more reasonable to study the saliva instead of dental plaque in this situation. A comprehensive and thorough investigation of the bacterial diversity of salivary microbiota is essential for understanding the how or whether *H. pylori* infection alters the salivary microbiota. The technology of high-throughput sequencing has provided new cognizance of the structures and compositions of microbiota communities.

By comparing the alpha diversity indexes we found that the bacterial diversity in saliva was similar among the *H. pylori* uninfected and *H. pylori* infected people. Our study is consistent with the notion that *H. pylori* in the stomach has little or no effect on the bacterial diversity of the oral cavity [20]. The Shannon diversity index, ACE richness index, and Chao 1 richness estimator all declined after eradication of *H. pylori* compared to the baseline samples (*p* < 0.05), which was consistent with the prior studies that use of PPI and antibiotics may affect the oral microbiome [21, 22].

According to the beta diversity analysis based on the unweighted UniFrac distances, the community structures of saliva microbiota were different in *H. pylori* uninfected and infected individuals, which was contrary to the results of Schulz’s study [20]. Samples from the *H. pylori* infected subjects tended to cluster together, while the microbiota in the uninfected subjects appeared to be more variable suggesting that gastric *H. pylori* infection may affect oral bacterial components. Clear segregations by the PCoA and NMDS analysis among individuals before and after *H. pylori* eradication therapy demonstrated that successful eradication or eradication therapy changed the oral bacterial components to some extent.

In addition to the presence of different bacterial members, the abundance of some bacteria also differed significantly among groups. We clearly observed that some bacteria in the saliva of *H. pylori* infected individuals showed a significantly reduced abundance, among which *Aggregatibacter*, *Klebsiella*, *Fusobacterium*, and *Parvimonas* species were pathogenic bacteria, causing infective endocarditis, liver abscess, pneumonia, meningitis, systemic infections, et al. [23–25]. However, the abundance of other bacteria significantly increased in saliva of *H. pylori* infected individuals, most of which were oral microbiota composition, including *Sphingomonas*, *Ochrobactrum*, *Afipia*, *Leptotrichia*, *Oribacterium*, and *Moryell*, except *Acinetobacter* species causing infectious diseases like pneumonia and urinary tract infections [26], and *Leptotrichia* species, a potential cariogenic bacteria [27]. While in Schulz’s study, no significant difference in oral communities between *H. pylori* infected and uninfected individuals was detected at genus level [21], this may be due to the different target sequencing region of 16 s rDNA, sample size, or geographic location. Interestingly, most *H. pylori*-enriched genera increased after the eradication, including *Ralstonia*, *Leptotrichia*, *Sphingomonas*, *Leptothrix*, *Oribacterium*, and *Acinetobacter.* The exception was *Ochrobactrum*. However, genera low expressed in *H. pylori* infected saliva experienced a further decline after *H. pylori* eradication therapy, including *Alloprevotella*, *Aggregatibacter*, *Leptotrichlaceae__G_1_*, *Parvimonas*, and *Fusobacterium*. Our study suggests that *H. pylori* infection may change the salivary microbiota, eradication therapy would further change the bacteria composition of saliva microbiota. Although the clinical significance of these alterations was not known, *H. pylori* unexpectedly and clearly altered the oral microbiota composition.

In our study, we found that *H. pylori* infection didn’t change the abundance and diversity, but changed the composition structure of salivary microbiome. *H. pylori* is colonized in the stomach, and there is a certain distance from stomach to the oral cavity. Even though *H. pylori* can cause a large change in the abundance of the microbiota in the stomach, it is difficult to affect the abundance of the salivary microbial flora. Previous studies have reported acid inhibition in upper gastric tract may have an effect on the oral microbiome leading to alterations in the microbiota [28]. In addition, changes in gastric pH could also lead to an alteration in the pH of oral cavity [29]. We proposed that *H. pylori* likely changed the community and structure of oral microbiota through changes in the acidic environment in stomach by generating large amount of urease, an enzyme which decomposes urea into ammonia and carbon dioxide and transiently reduce the acidic environment in the stomach [30], leading to the further changes in pH of oral cavity. The use of PPIs during the eradication therapy would further inhibit the pH in stomach, leading to further alteration in saliva microbiota, which can partially explain why genera enriched in *H. pylori* infected individuals would further increase and genera low expressed in *H. pylori* infected individuals would decline after successful eradication. Although the precise mechanism has yet to be clarified, to our knowledge this is the first study to clearly show oral microbiota alterations as a result of *H. pylori* infection in a cohort of subjects. Additional studies like metagenomics sequencing or metabolomics to investigate these possible causal relationships would likely provide interesting findings.

Using amplicon sequencing of 16S rDNA V3-V4 hypervariable regions we detected *H. pylori* in the oral cavity of almost half of the subjects regardless of whether they had gastric infection with *H. pylori*. Subjects who did not have *H. pylori* in the oral cavity before eradication surprisingly had *H. pylori* detected in saliva samples after *H. pylori* eradication therapy. Clearly, using these techniques the prevalence of *H. pylori* in oral cavity is not clearly associated with colonization status in the stomach which is not consistent with the notion that the oral cavity represents a secondary site for *H. pylori* colonization [31]*.* The gastric and oral mucosa differ markedly. For example, of the two only the gastric mucosa expresses Lewis^b^ antigen, an ABO blood group antigen, which enables adherence of *H. pylori* to the epithelial surfaces. It has been proposed that *H. pylori* is a passerby in oral cavity, rather than a colonizer and it may be also be included in the material in gastroesophageal reflux. The natural history of *H. pylori* infection has been that after *H. pylori* eradication from the stomach, gastric reinfection is rare and when it occurs early it can often be shown to be recrudescence (the same genotype) whereas later reinfections are most often reinfection with a different genotype [32]. The hypothesis that *H. pylori* was a common passerby rather than a colonizer would partly explain why recurrences are most common in areas with poor sanitation and a high prevalence of *H. pylori* and rare in developed countries whose frequency of *H. pylori* infection had become much lower than that of poor regions.

### Strengths and limitations

Our study showed that *H. pylori* infection and the eradication treatment resulted in alterations of oral microbiota. However, there were limitations to our study. The technique of high-throughput sequencing we used in our study could not detect *H. pylori*-specific virulence factors such as VacA, CagA, OipA, etc. or full sequence [33]. Although the samples of the first set of cross-sectional analysis were collected at different times in August 2018, the collection interval between the first and the last collection were equal. All the samples underwent DNA extraction and sequencing analysis at the same time to make our result were comparable. One issue with the interpretation is that there was no control sample of *H. pylori* uninfected individuals receiving the same antimicrobial therapy which precluded determination about whether the presence of *H. pylori*, the antimicrobial therapy, or both were dominant factors in changing the within-individual diversity of the oral cavity.

## Conclusions

Our study using high-throughput sequencing showed that *H. pylori* is present commonly in the oral cavity with no clear relation to *H. pylori* infection of the stomach. Both *H. pylori* infection and eradication therapy caused alterations in community and structure of the oral microbiota.

## Supplementary information

**Additional file 1: Table S1**. Clinical parameters of the 58 subjects. N = uninfected group, P = infected group, PE = pre-eradicated group, E = eradicated group.

**Additional file 2: Table S2**. Barcodes of all samples subjected to multiplex sequencing. N = uninfected group, P = infected group, E = eradicated group.

**Additional file 3: Table S3**. Sequences and modified OTUs of all saliva samples from sequencing at 97% identity. N = uninfected group, P = infected group, E = eradicated group.

**Additional file 4: Figure S1**. Length distribution of sequences determined by Illumina MiSeq sequencing.

**Additional file 5: Table S4**. Modified OTU table at 97% identity. N = uninfected group, P = infected group, E = eradicated group.

**Additional file 6: Figure S2**. A classification tree showing bacterial abundance by MEGAN. The taxonomy compositions and abundances were visualized by MEGAN (version 6.6.7). The larger the area of the colored pie chart, the greater the bacterial abundance. Different colors represent different groups, and the larger the colored sectorial area at a branch, the more the corresponding group contributed to the bacterial abundance. N = uninfected group, P = infected group, E = eradicated group.

**Additional file 7: Figure S3**. Alpha diversity (observed species number) among groups. **A.** N group and P group showed similar alpha diversity (*p* > 0.05). N = uninfected group, P = infected group. **B.** The observed species in E group were significantly lower than that of PE group and N group (*p* < 0.01); One asterisk indicates significant differences (*p* < 0.05, Student’s t-test), two asterisk indicates p < 0.01, three asterisk indicates *p* < 0.001. N = uninfected group, PE = pre-eradicated group, E = eradicated group.

**Additional file 8: Figure S4**. Rank abundance curves for all OTUs. N = uninfected group, P = infected group, E = eradicated group.

**Additional file 9: Figure S5**. Nonmetric Multidimensional Scaling (NMDS) based on unweighted UniFrac distances at the OUT level at 97% identity. Each sample is represented by a dot. A. The samples formed well-separated clusters corresponding to the two groups, suggesting that the bacterial structures in N group and P group were different. N = uninfected group, P = infected group. Red squares represent the N samples. Blue triangles represent the P samples. **B.** Blue triangles represent the N samples. Red circles represent the E samples. The samples formed well-separated clusters corresponding to the three groups, suggesting that the bacterial structures in E group, PE group, and N group were different. N = uninfected group, PE = pre-eradicated group, E = eradicated group.

**Additional file 10: Figure S6**. Comparison of microbial variations at the genus level, using the LEfSe online tool. **A.** Histogram of the LDA scores for differentially abundant features among groups. The threshold on the logarithmic LDA score for discriminative features was set to 2.0. N = uninfected group, PE = pre-eradicated group, E = eradicated group. **B.** Cladogram for taxonomic representation of significantly differences among groups. Differences are represented in the color of the most abundant taxa (red indicating N group, blue indicating PE group, green indicating E group, and white indicating non-significant). N = uninfected group, PE = pre-eradicated group, E = eradicated group.

## Data Availability

The datasets analysed during the current study are available in the NCBI Sequence Read Archive at https://www.ncbi.nlm.nih.gov/ under accession number SRP167714. The study protocol and statistical analysis plan are available online from https://clinicaltrials.gov/ct2/show/ NCT03730766.

## Abbreviations

*H. pylori*: *Helicobacter pylori*
OTUs: Operational taxonomic units
PCR: Polymerase chain reaction
QIIME: Quantitative Insights Into Microbial Ecology
HOMD: Human Oral Microbiome Database
ACE: Abundance-based Coverage Estimator
PCoA: Principal coordinate analysis
NMDS: Nonmetric Multidimensional Scaling
LEfSe: Linear discriminant analysis effect size
^13^C-UBT: ^13^C urea breath test
RUT: Rapid urease test
PPI: Proton pump inhibitor
SD: Standard Deviation
N: Uninfected group
P: Infected group
E: Eradicated group
PE: Pre-eradicated group
