# Supplementary figures and images for: Analysis of by high-throughput sequencing: Helicobacter pylori infection and salivary microbiome
Source: BMC Oral Health. 2020 Mar 20;20:84. doi: 10.1186/s12903-020-01070-1 (PMC7333272; doi:10.1186/s12903-020-01070-1)

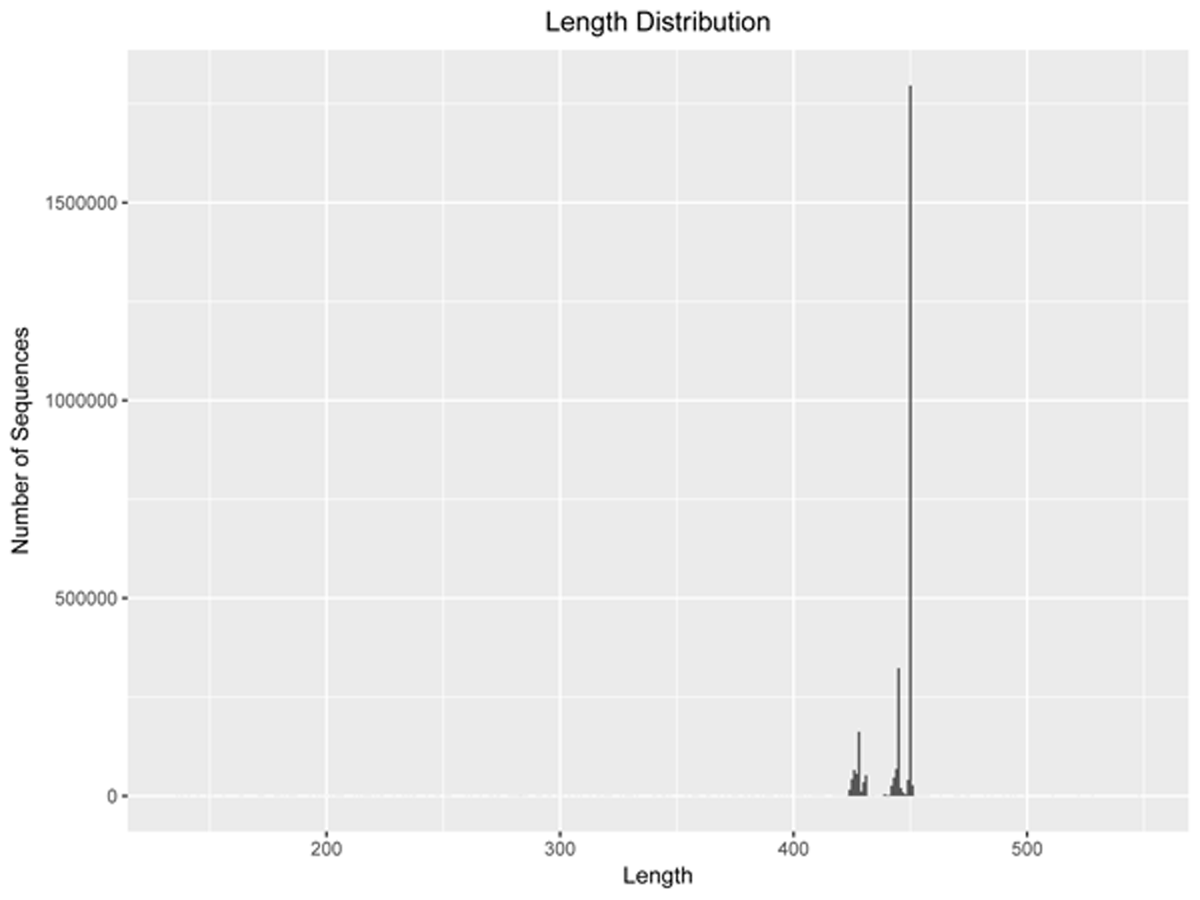

Supplement: Supplementary file 4 — Additional file 4: Figure S1. Length distribution of sequences determined by Illumina MiSeq sequencing. [file 12903_2020_1070_MOESM4_ESM.tif]

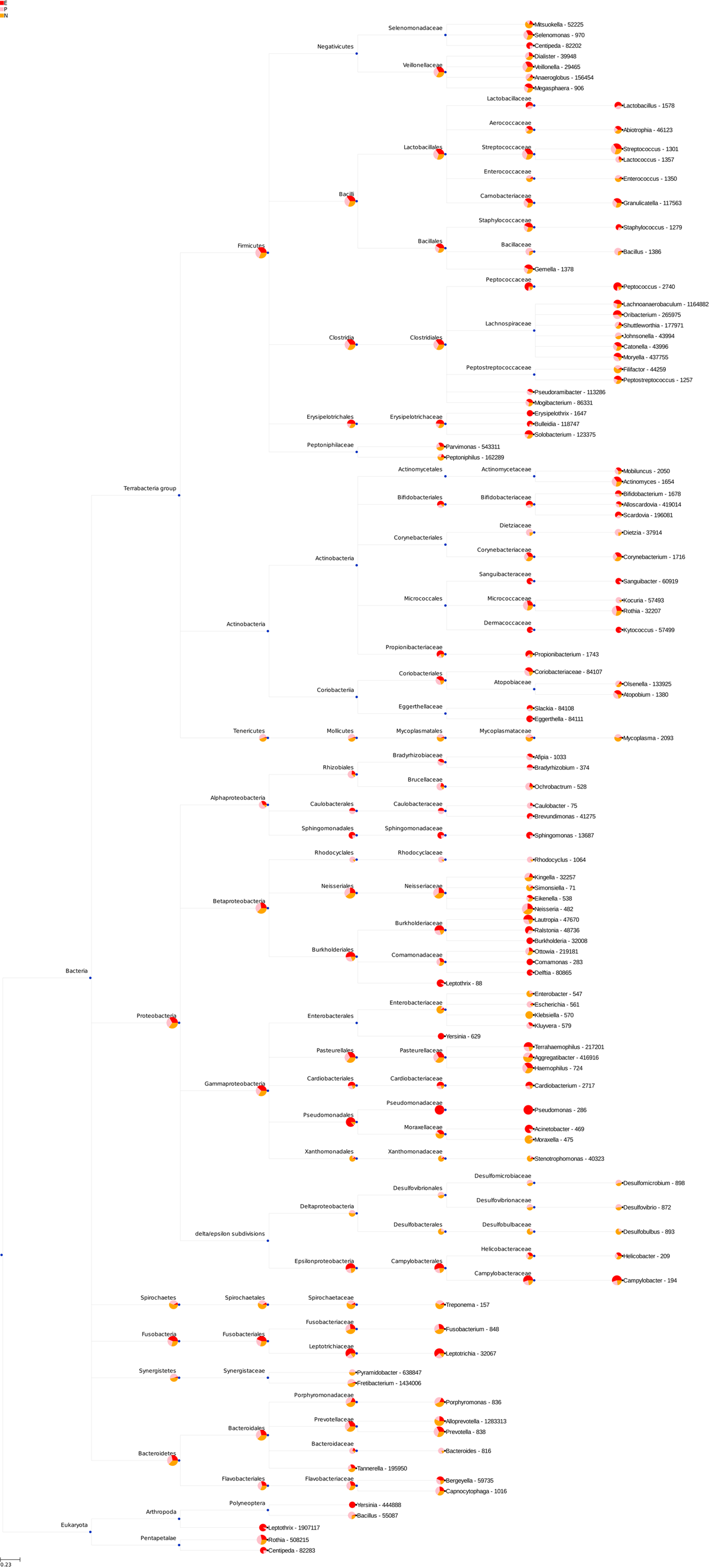

Supplement: Supplementary file 6 — Additional file 6: Figure S2. A classification tree showing bacterial abundance by MEGAN. The taxonomy compositions and abundances were visualized by MEGAN (version 6.6.7). The larger the area of the colored pie chart, the greater the bacterial abundance. Different colors represent different groups, and the larger the colored sectorial area at a branch, the more the corresponding group contributed to the bacterial abundance. N = uninfected group, P = infected group, E = eradicated group. [file 12903_2020_1070_MOESM6_ESM.tif]

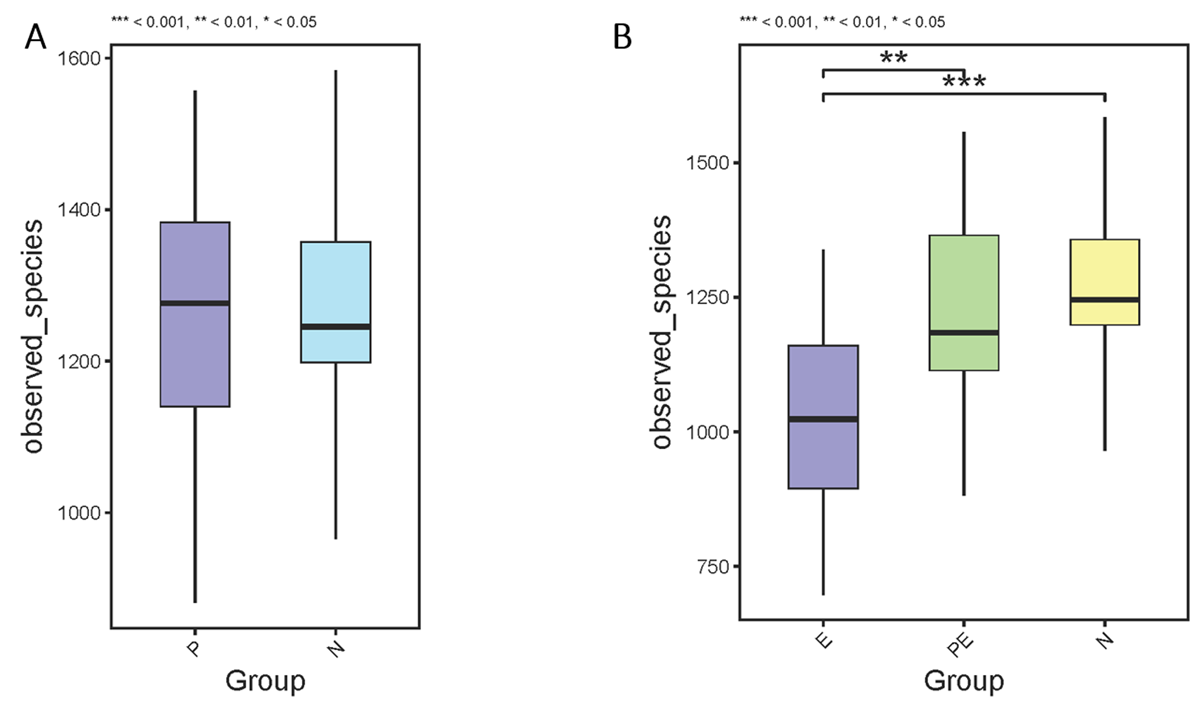

Supplement: Supplementary file 7 — Additional file 7: Figure S3. Alpha diversity (observed species number) among groups. A. N group and P group showed similar alpha diversity (p > 0.05). N = uninfected group, P = infected group. B. The observed species in E group were significantly lower than that of PE group and N group (p < 0.01); One asterisk indicates significant differences (p < 0.05, Student’s t-test), two asterisk indicates p < 0.01, three asterisk indicates p < 0.001. N = uninfected group, PE = pre-eradicated group, E = eradicated group. [file 12903_2020_1070_MOESM7_ESM.tif]

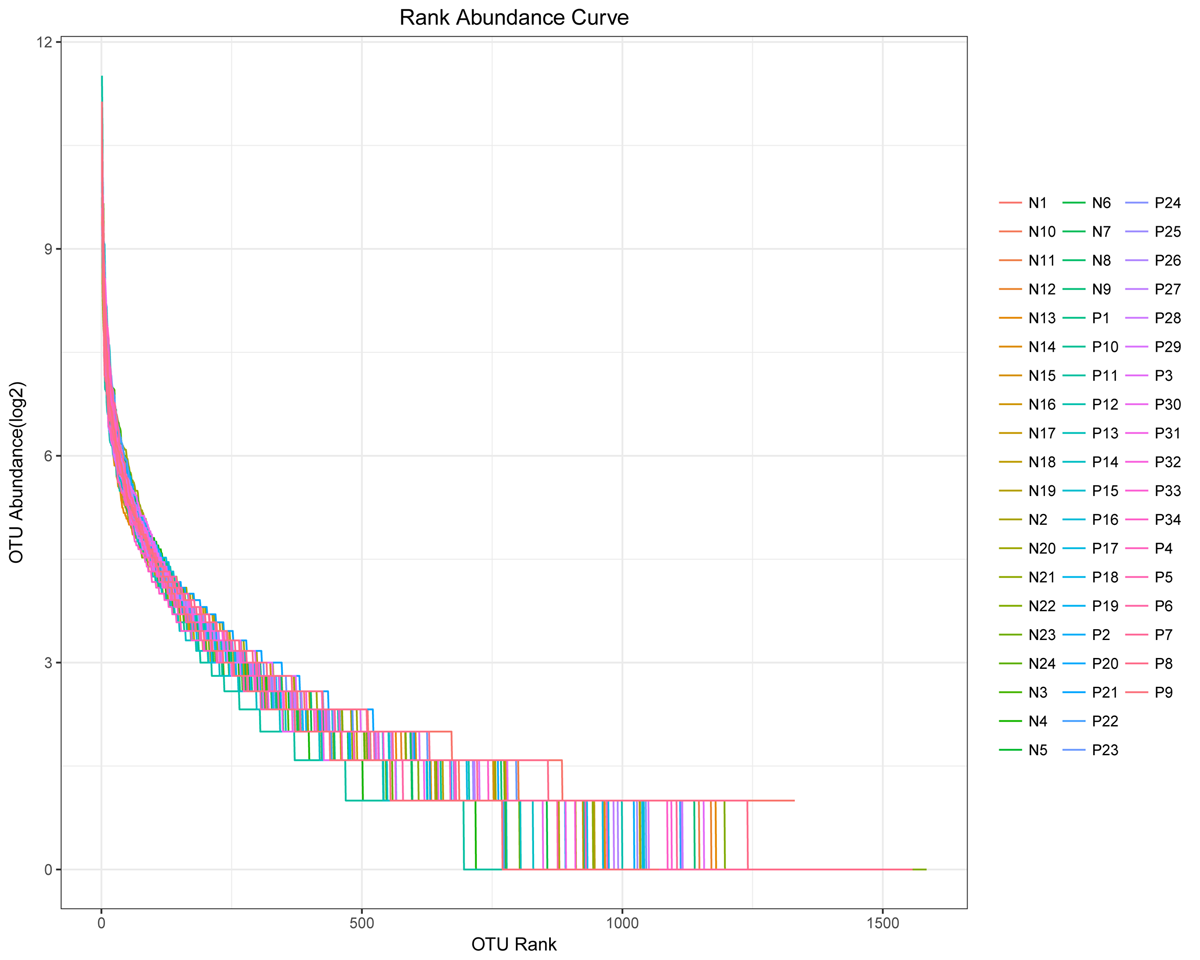

Supplement: Supplementary file 8 — Additional file 8: Figure S4. Rank abundance curves for all OTUs. N = uninfected group, P = infected group, E = eradicated group. [file 12903_2020_1070_MOESM8_ESM.tif]

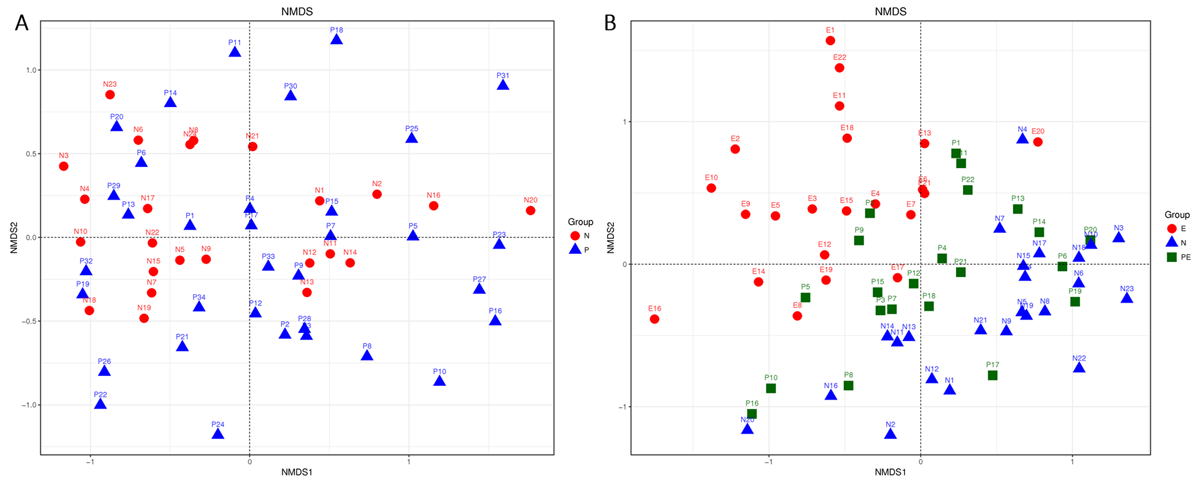

Supplement: Supplementary file 9 — Additional file 9: Figure S5. Nonmetric Multidimensional Scaling (NMDS) based on unweighted UniFrac distances at the OUT level at 97% identity. Each sample is represented by a dot. A. The samples formed well-separated clusters corresponding to the two groups, suggesting that the bacterial structures in N group and P group were different. N = uninfected group, P = infected group. Red squares represent the N samples. Blue triangles represent the P samples. B. Blue triangles represent the N samples. Red circles represent the E samples. The samples formed well-separated clusters corresponding to the three groups, suggesting that the bacterial structures in E group, PE group, and N group were different. N = uninfected group, PE = pre-eradicated group, E = eradicated group. [file 12903_2020_1070_MOESM9_ESM.tif]

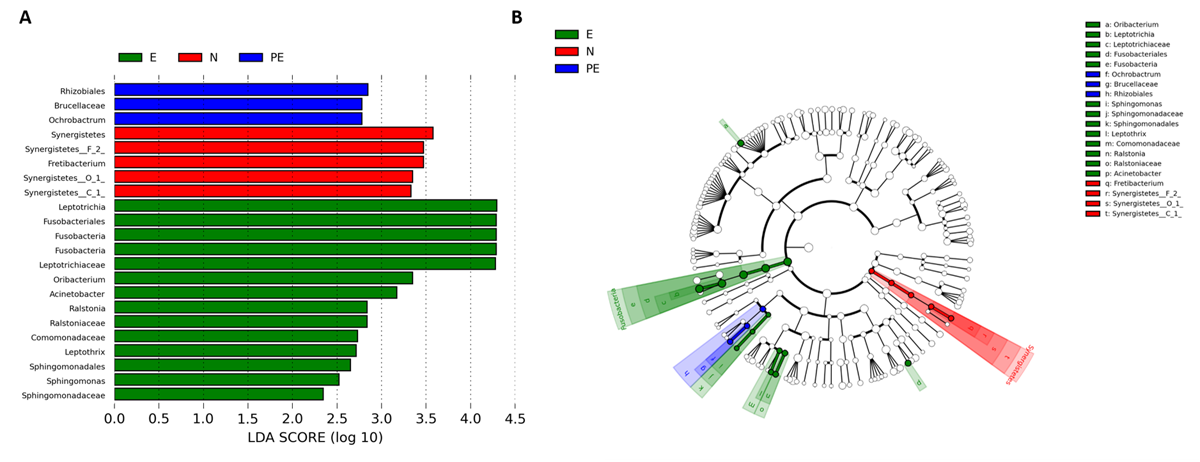

Supplement: Supplementary file 10 — Additional file 10: Figure S6. Comparison of microbial variations at the genus level, using the LEfSe online tool. A. Histogram of the LDA scores for differentially abundant features among groups. The threshold on the logarithmic LDA score for discriminative features was set to 2.0. N = uninfected group, PE = pre-eradicated group, E = eradicated group. B. Cladogram for taxonomic representation of significantly differences among groups. Differences are represented in the color of the most abundant taxa (red indicating N group, blue indicating PE group, green indicating E group, and white indicating non-significant). N = uninfected group, PE = pre-eradicated group, E = eradicated group. [file 12903_2020_1070_MOESM10_ESM.tif]
